# Guidelines for Performing CRISPR/Cas9 Genome Editing for Gene Validation and Trait Improvement in Crops

**DOI:** 10.3390/plants12203564

**Published:** 2023-10-13

**Authors:** Nikolaos Tsakirpaloglou, Endang M. Septiningsih, Michael J. Thomson

**Affiliations:** Department of Soil and Crop Sciences, Texas A&M University, College Station, TX 77843, USA; n.tsakirpaloglou@ag.tamu.edu (N.T.); eseptiningsih@tamu.edu (E.M.S.)

**Keywords:** genome editing, crop improvement, high-throughput pipeline, CRISPR/Cas9

## Abstract

With the rapid advances in plant genome editing techniques over the past 10 years, more efficient and powerful crop genome editing applications are now possible. Candidate genes for key traits can be validated using CRISPR/Cas9-based knockouts and through the up- and down-regulation of gene expression. Likewise, new trait improvement approaches can take advantage of targeted editing to improve stress tolerance, disease resistance, and nutritional traits. However, several key steps in the process can prove tricky for researchers who might be new to plant genome editing. Here, we present step-by-step guidelines and best practices for a crop genome editing pipeline that should help to improve the rate of success. Important factors in the process include proper target sequence analysis and single guide RNA (sgRNA) design, sequencing of the target site in the genotypes of interest, performing an in vitro CRISPR/Cas9 ribonucleoprotein (RNP) assay to validate the designed sgRNAs, preparing the transformation constructs, considering a protoplast editing step as further validation, and, finally, stable plant transformation and mutation detection by Sanger and/or next-generation sequencing. With these detailed guidelines, a new user should be able to quickly set up a genome editing pipeline in their crop of interest and start making progress with the different CRISPR/Cas-based editing variants for gene validation and trait improvement purposes.

## 1. Introduction

Looming threats to global food security and a growing need for improved human nutrition justify increasing investments in next-generation technologies to accelerate progress in crop improvement. However, new paradigms are needed to efficiently translate genomic research discoveries into accelerated crop improvement programs to address future challenges. A new generation of plant breeding technologies, including genomic selection, genome editing, and, ultimately, controlled genetic recombination, will be required to address future challenges in developing more nutritious foods and climate-resilient crops [1]. Of these new technologies, genome editing offers an unprecedented opportunity to rapidly characterize and deploy key genes and alleles for crop improvement [2].

In 2012, a landmark discovery demonstrated that Clustered Regularly Interspaced Short Palindromic Repeat (CRISPR)-based bacterial defense systems can be used for precise genome editing based on a single guide RNA (sgRNA) and the nuclease Cas9 [3]. Since then, CRISPR technologies have begun to transform the fields of medicine, microbiology, and plant and animal research [4]. Massive investments in CRISPR technologies for medical research have begun to spill over into the plant sciences, and new insights, discoveries, and applications are being published weekly. Genome editing offers a promising approach to rapidly accelerate plant breeding efforts by precisely modifying genes or genome areas of interest [5]. The regulatory policies for genome-edited products vary by jurisdiction but may be straightforward in some cases [6,7,8,9,10,11].

**Figure 1:**
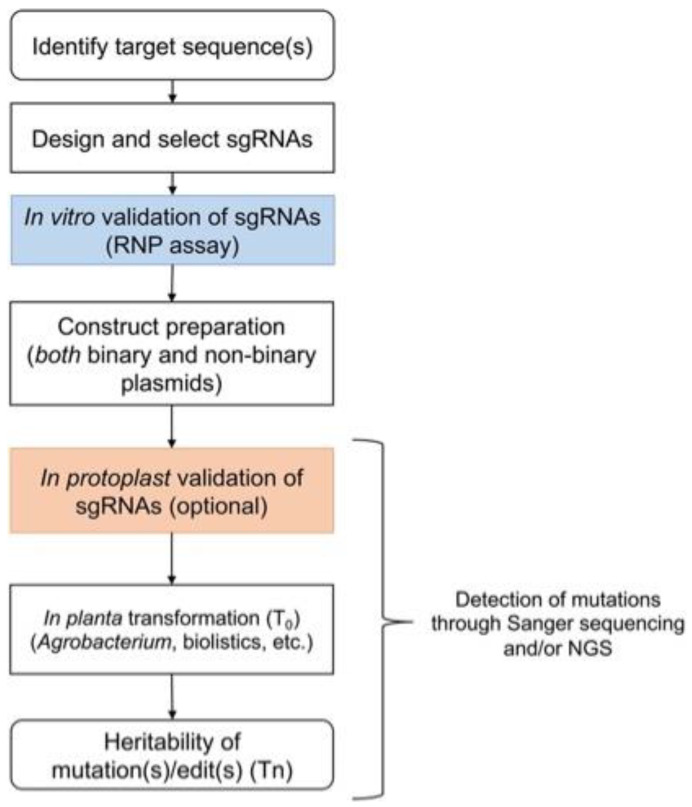
A stepwise approach for genome editing in various crop species. A series of different steps is followed for the selection of target sequences, the design of sgRNAs, and their validation in vitro or with protoplasts before stable transformation. Detection of the putative CRISPR/Cas9-mediated edits can be performed using Sanger sequencing or next-generation sequencing in T_0_ and subsequent generations. It is important to note that as the development pipeline primarily uses transgenic modification as a step in the production of genome-edited plants, the need to remove such modifications is a universal prerequisite for the different jurisdictions [10,16,17].

The identification of causal genes controlling key traits in crop species often begins with quantitative trait locus (QTL) mapping and genome-wide association studies (GWASs). Subsequently, a list of candidate genes is refined, and functional validation is performed through complementation and/or genome editing [12]. Recent advances in genome editing techniques have begun to greatly accelerate progress in the genetic dissection of key traits in plants but have not yet been thoroughly optimized for crop improvement [13]. Although the greatest impact of CRISPR/Cas-based techniques currently lies in functional gene validation, more opportunities will arise for trait improvement as the technology matures and the regulatory framework moves towards facilitating the commercialization of gene-edited products in certain jurisdictions [8,9,10,11,14]. The past 10 years have seen rapid progress in CRISPR/Cas-based genome editing in plants, and it is a good time to take stock of the lessons learned and to review the latest advances for efficient genome editing in crop plants. Here, we present guidelines and recommended best practices for a step-by-step approach for the development and optimization of a genome editing pipeline for different crop species (Figure 1). Although these guidelines are currently best suited for gene validation studies, in the future, we expect that the outputs of this pipeline could fit into the conventional breeding pipeline to further advance and improve various crops for different traits [15].

## 2. Procedure

### 2.1. In Silico Sequence Analysis

After selecting the target candidate genes for editing (as previously discussed in [7]), the first steps are to obtain the target sequences, including genomic DNA, mRNA, and coding sequence (CDS), from online sources and to confirm the target gene structure through multiple sequence alignments. Although predicted gene annotations can be useful to get started, they are not always reliable, and confirming the gene structure manually helps to provide better results. The precise locations of the start codon and the gene’s introns and exons are required to help ensure the successful selection of the desired target sequences and the subsequent design of sgRNAs, depending on the goals of the experiment. For example, disrupting (knocking out) gene function is most efficiently achieved by designing sgRNAs in an exon near the 5′-end of the gene. This increases the likelihood of generating frameshift mutations that lead to premature stop codons and truncated proteins. It is important to note that the inclusion of alternative splicing variants is important at the transcript and/or coding sequence level since this will impact exon/intron boundaries.

1. Identify the target sequences of the crop species of interest from their respective databases (Table 1).
2. Download the genomic sequence of interest and the respective transcript and coding sequences.

*Note*: Ensure that the transcript and coding sequences of all the predicted splicing variants are downloaded.

3. Map the obtained transcript and coding sequences to the genomic sequence to prepare the gene structure for the respective gene, including intron/exon locations for all transcript variants.

*Note*: In our case, we used MAFFT Multiple Sequence Alignment Software Version 7 [18] through Benchling (https://www.benchling.com/ (accessed on 1 September 2023)).

4. Annotate the target genomic sequence manually or confirm the gene annotation found in various sequence databases.

### 2.2. Design of Guide RNAs

**Figure 2:**
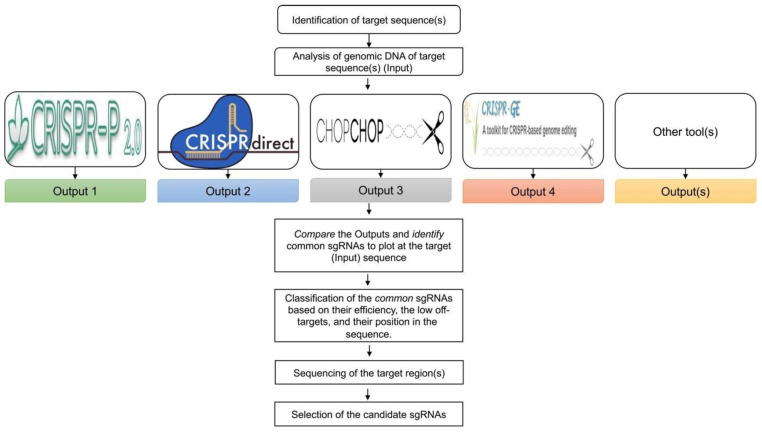
Design of sgRNAs. Analysis of the genomic DNA sequence of the respective target(s) can be performed through different web tools that are commonly used for the design of sgRNAs in plants. Comparison of the output of these databases results in the identification and selection of sgRNAs present in all the outputs. Classification of the common sgRNAs can be performed based on their efficiency, fewer potential off-target sites, and their position in the sequence. Before the final selection of the sgRNAs, sequencing of the target regions in the sgRNA region is recommended to ensure consistency between the in-silico and the actual cultivar sequence.

The purpose of this step is to design and select sgRNAs that will create the desirable edits in the target areas of the genome with high efficiency. Currently, there are many online software tools (online software without the need to be installed) for the design of sgRNAs for different crop species (Figure 2). All these tools enable the design of sgRNAs using different parameters and algorithms. Interestingly, there is significant variation between the output of these tools for the same input sequence, leading to questions regarding which tool or algorithm provides the most accurate classification for the design and selection of sgRNAs. Hence, the best practice is to compare the output of multiple online tools to identify the sgRNAs present in all outputs, assuming that the selection of the specific sequences using multiple tools/algorithms implies optimal design and efficiency and therefore a greater chance of success. Mapping the “common” sgRNAs onto the genomic sequence often reveals that most of these sgRNAs are clustered in specific areas of the target gene. In addition to selecting sgRNAs that are common across the design tools, additional characteristics of the obtained sgRNAs, such as predicted efficiency, a lower probability for off-target modifications, and the sequence position for the creation of mutations, help to increase the chance of successful sgRNAs. Note that a previous study suggested that the sgRNA rankings using the current set of online design tools are not entirely predictive in plants, since most of the algorithms were trained using animal systems [19]. Therefore, the best practice would be to design multiple gRNAs per target, so that there is a backup if one of the sgRNA designs fails. This also allows for simple size selection when screening PCR amplicons for a double mutant event, which would lead to a deletion of the segment between the two sgRNAs.

5. Use the genomic sequence of the target region as an “input sequence” for the analysis in online databases commonly used for the design of sgRNAs in plants (Table 2), such as CRISPR-P 2.0 [20], CRISPR-direct [21], CHOPCHOP [22], target design [23], CRISPR-PLANT v2 [24], and/or other available web tools [25], focusing on the design of sgRNAs.

*Note*: In our experiments, we observed that the output of this analysis from the respective databases differed substantially for the same target region(s).

All these databases integrate genome data from several crop species for the design of sgRNAs; however, if the desired species is not available, a close relative species could be utilized as the template.

6. Identify the “common” sgRNAs that are present in the output of most, if not all, databases.

*Note*: In our case, we found that most of the common sgRNAs were often clustered at certain, potentially conserved, regions of the target sequence.

7. Map the selected sgRNAs on the gene structure alignments and classify them based on the following criteria:
    i. Targeting all the transcript variants (pay attention to alternative splicing);
   ii. Predicted high efficiency;
  iii. Fewer off-targets;
  iv. For knockout mutations, create the intended mutations near the beginning of the coding sequence to generate premature stop codons and truncated peptides.

### 2.3. Primer Design and Sequencing of Target Regions

It is well documented that allelic variations within a genome of the same species, such as insertions/deletions (InDels) and single nucleotide polymorphisms (SNPs), occur quite frequently [26]. It is therefore imperative to ensure that the actual target sequences of the selected sgRNA regions (see Section 2.2) are identical to those obtained electronically based on the reference genome, before sgRNA selections are finalized, to ensure their proper functioning in the respective target region(s). For this purpose, sequencing of the target region is recommended, preferably using PCR with proofreading Taq polymerase and TOPO cloning.

8. Design primers that flank the target regions using NCBI Primer Blast [27] and Oligo Analyzer (IDT) (https://www.idtdna.com/calc/Analyzer/Home/Instructions (accessed on 1 September 2023)).

*Note*: The ideal target amplicon size should fall between 500 bp and 1200 bp to yield the best results from Sanger sequencing. However, the size of the amplicon may vary, especially in cases of polyploid crops, where it is necessary to design genome-specific primers.

The target site(s) of the sgRNA(s) should be positioned at various locations within the target region, with a minimum distance of ≥150 bp from the respective primers, avoiding the exact center of the amplicon so that multiple bands can be easily visualized when cleaving the target site with the in vitro RNP assay (see Section 2.4 below).

9. Amplify the target region using proofreading Taq polymerase.

*Note*: Various providers have different enzymes with proofreading activities. Some of the commonly used enzymes include Phusion High-Fidelity DNA Polymerase (2 U/µL) (Thermo Fisher Scientific, Waltham, MA, USA), Q5 High-Fidelity DNA Polymerase (NEB), and KAPA HiFi DNA Polymerase (Roche, Indianapolis, IN, USA).

10. Agarose electrophoresis and excision of the target fragments from the gel, followed by purification with a gel extraction kit, enables the collection of clean amplicons with minimal impurities.
11. Sanger sequencing of the obtained amplicon can be performed at this point; however, TOPO-cloning (for example using the Zero Blunt™ TOPO™ PCR Cloning Kit, Thermo Fisher Scientific, Waltham, MA, USA) of the fragments and submission of 3–5 clones derived from each amplicon for sequencing is highly recommended, especially when sequencing is intended for genotyping the target region. This ensures that all possible sequence variants in the amplicon are detected.

### 2.4. In Vitro CRISPR/Cas9 Ribonucleoprotein (RNP) Assay Validation Step 1

The in vitro cleavage of DNA amplicons using the CRISPR/Cas9 RNP complex can serve as a useful validation step to assess the functionality and relative efficiency of the CRISPR system for the desirable targets [28,29]. For this purpose, the procedure described on the NEB website (https://www.neb.com/protocols/2014/05/01/in-vitro-digestion-of-dna-with-cas9-nuclease-s-pyogenes-m0386, accessed on 1 September 2023) and the associated protocol from protocols.io (https://dx.doi.org/10.17504/protocols.io.be6fjhbn, accessed on 1 September 2023) for the in vitro digestion of DNA with Cas9 nuclease can be followed. In short, a purified/eluted PCR amplicon flanking the target site (used in Section 2.3. above) is incubated with synthetic sgRNAs and purified Cas9 nuclease. This is then run on gel electrophoresis to distinguish the cut (i.e., successfully cleaved by the sgRNA/Cas9 RNP complex) vs. uncut (i.e., not cleaved by the RNP complex) bands, which indicates the level of efficiency of Cas9 to cleave the target site with those specific sgRNA designs.

12. Perform the assay using the amplicon that was eluted from step 11 as substrate DNA.

*Notes*: In our experience ≥70 ng of substrate DNA is adequate for clear visualization in the agarose gel.

The sgRNAs can be synthesized and obtained from Synthego Corporation, Redwood City, CA, USA (https://www.synthego.com, accessed on 1 September 2023), whereas Cas9 nuclease can be obtained commercially from NEB (Catalog No.: M0386), Sigma-Aldrich, Burlington, MA, USA (Catalog No.: CAS9PROT), Thermo Fisher (Catalog No.: A36499), or other companies.

13. Visualization of the fragments can be performed using agarose electrophoresis (generally 1.5–2% (*w*/*v*)), at 100 V for 20–45 min, depending on the size of the expected fragments. A single uncut band indicates failure of the sgRNA with that target amplicon, whereas a larger uncut band combined with two smaller cut bands (often quite faint) indicates successful cleavage by Cas9 with those sgRNA designs.

### 2.5. Construct Preparation

Multiple modular approaches have been proposed by different research groups for plasmid preparation [30,31,32,33]. Most of them are based on the utilization of type IIS restriction enzymes and subsequent Golden Gate cloning for the assembly of the different modules. The flexibility that these systems offer in terms of combining different components enables the consideration of several approaches for construct design and development, depending on the target crop, as well as the overall size of the plasmid. Moreover, several RNA polymerase III (Pol-III)-driven and Pol-II-driven systems have been widely employed for efficient in planta expression of multiplexed sgRNA systems [5,14]. The utilization of the endogenous cell machinery for precise processing and the efficient production of multiple sgRNAs has also been quite successful [34,35].

14. Assemble binary and/or non-binary version(s) of the plasmids, utilizing the different components of the previously described modular systems.

*Note*: Consider the use of codon-optimized Cas9 nucleases for monocot or dicot species, depending on the target crop.

The utilization of tRNA-sgRNA and cys4 systems is recommended for the expression of multiple sgRNAs from a single promoter in different crop species [31]. All relevant plasmid constructs for CRISPR-Cas9 techniques in plants are publicly available, along with detailed information, from the Addgene repository (www.addgene.org, accessed on 1 September 2023).

### 2.6. Protoplast Transformation and Validation of sgRNAs (Validation step 2; Optional)

The development of a robust and reproducible protoplast isolation method enables the rigorous evaluation of genome editing components before stable transformation, as well as the potential for protoplast regeneration in several crop species [36,37,38,39]. For this purpose, we tried to establish a systematic approach in our lab for the isolation and transformation of protoplasts for both monocot and dicot crop species [40,41] to serve as a platform for assessing the effectiveness of our constructs to generate induced mutations at the target sites.

15. Isolate protoplasts from the respective crop species.

*Note*: A comprehensive list of protoplast isolation protocols for different plant species can be found in Reed and Bargmann, 2021 [38].

16. Optimize the length of the viability of the isolated protoplasts [42] at different temperatures (e.g., 4 °C, 13 °C, and 25 °C).

*Note*: To determine the viability of the protoplasts, we used the Plant Cell Viability Assay Kit (Sigma-Aldrich, Burlington, MA, USA, Cat. No.: PA0100-1KT).

17. Optimize the polyethylene glycol (PEG) concentration [43,44] to enable the efficient transformation of isolated protoplasts using a non-binary vector that contains a constitutive promoter to drive GFP expression.
18. Optimize the plasmid concentration required for efficient protoplast transformation by testing different concentrations of the previously utilized GFP plasmid (see step 17).
19. Assess the GFP expression levels when driven by promoters that are used to drive the expression of different biological components of the designed genome editing cassette (if needed).
20. Transform protoplasts using the non-binary version of the cassette containing Cas9 nuclease and the sgRNAs.

Perform DNA extraction and screening to detect mutations to determine the efficacy of the tested sgRNAs (see Section 2.8).

### 2.7. Stable Transformation of Crop Species for Efficient Genome Editing

For the efficient delivery and expression of biological and/or genome editing components, several plant transformation strategies have been considered [45,46,47]. Agrobacterium-mediated transformation remains the popular choice for plant transformation and the intact integration of large DNA fragments into plant chromosomes [48]. However, limitations in the range of plant host genotypes that are competent for Agrobacterium infection, combined with lengthy regeneration procedures for many crop species, create a bottleneck that often prohibits high-throughput transformation and genome editing [45]. Nonetheless, Agrobacterium-mediated transformation is still the method of choice for most genome editing experiments. Particle bombardment is also considered a popular approach for nuclear plant transformation; however, the detection of multiple integration sites and the high frequency of random rearrangements of the integrated copies, which can lead to unpredictable effects in the events generated using this approach, present further challenges for their downstream analysis and application [45]. The biolistic delivery of in vitro transcripts or ribonucleoprotein complexes of CRISPR/Cas9 has also been applied recently in several crop species to preclude genome integration effects and to potentially facilitate downstream commercial applications [49,50,51,52]. However, these alternate biolistic approaches have very low efficiencies and therefore require very large numbers of transformed explants to be successful.

Since *Agrobacterium*- and biolistic-based transformation methods require lengthy and labor-intensive in vitro tissue culture steps to regenerate whole plants from transformed explants or callus material, alternative approaches for in planta transformation have been explored. Nanoparticle-mediated gene transformation strategies for plant genetic engineering constitute an attractive strategy that could bypass the hurdles of tissue culture [53]. Recently, we were able to confirm carbon nanotube-mediated plasmid DNA delivery in rice leaves and seeds [54]. Despite the low efficiency detected in the editing of germinating rice seeds, the passive absorption and expression of plasmids attached to carbon nanotubes present an intriguing avenue for further exploration and optimization. At the same time, pollen magnetofection, a novel transformation methodology that involves the attachment of recombinant DNA to magnetic nanoparticles capable of penetrating pollen grains in a magnetic field [55,56], has not yet proven successful [57].

Virus-based RNA delivery systems have also attracted attention recently for the in planta delivery of genome editing components and the subsequent detection of mutations without the need for transgene integration and tissue culture. Multiplex genome editing has been achieved in tobacco using virus-based RNA delivery of the entire CRISPR/Cas9 cassette; however, the detected mutations were eliminated in subsequent generations of progeny lines, indicating a loss of transmissibility [58]. Alternatively, high editing efficiency was observed in wheat and maize when the viral system relied on infections of previously transgenic plants that overexpressed the Cas9 nuclease [59,60], further highlighting the potential of this approach despite limitations because of the narrow host range and other physical constraints.

Recent developments in the utilization of specific morphogenic factors for reprogramming somatic cells into embryonic ones hold the promise of improving crop transformation, particularly for monocot and recalcitrant species, or even eliminating the need for the tissue culture step for dicot species [45]. Moreover, the concurrent expression of a single growth regulator or more growth regulators, in combination with plasmids containing genome editing components, was shown to promote the development of stably transformed crops [61,62,63,64,65,66,67]. In addition, morphogenic factors improved the Agrobacterium-mediated transformation of maize and sorghum seedling leaf bases, enabling the direct and rapid formation of transformed plant embryos, which highlights the potential advantages of using leaf bases as the starting explant for maize and sorghum transformation and genome editing [68]. Nevertheless, despite recent advances in the utilization of morphogenic factors to accelerate crop transformation, there are still challenges in the development of efficient and reliable transformation systems that find widespread adoption, particularly for monocot species. Moreover, tissue and genotype dependency still hinder the widespread adoption of transgenic and genome editing technologies in many economically important species.

21. Stable transformation of the genome editing components using the desirable method.
22. Detection of mutations as described below (see Section 2.8).

### 2.8. Detection of Mutations

To readily detect the induced mutations generated using CRISPR/Cas application, several approaches could be considered. The restriction enzyme (RE) site loss method involves targeting a previously selected RE site within the sequence of interest that is proximal to the protospacer adjacent motif (PAM) [69]. It is well established that the Cas9 nuclease introduces a blunt cut three nucleotides from the PAM. Therefore, the selection of a DNA target with an overlapping restriction site near the PAM can result in disruption of the RE site during the repair process of the double-strand break via the error-prone non-homologous end joining pathway. Mutations can be detected by amplification of the target site and subsequent restriction digestion of the obtained amplicon with an appropriate RE, followed by gel electrophoresis (i.e., cut amplicons have an intact RE site and are not edited, whereas uncut bands indicate a disrupted RE site and therefore a successful edit). The sensitivity of this assay could be improved by digesting the template DNA restriction enzyme and subsequent PCR amplification [69,70]. The Surveyor assay, which identifies mismatches in the target amplicon due to edits, constitutes an alternative approach for the detection of induced mutations at the target site(s) due to genome editing [71,72,73]. Although it is theoretically applicable to any target sequence, the RE site loss approach is less sensitive because it requires a higher mutagenesis rate [69]. In any case, both approaches have prerequisites that make them cumbersome to fully utilize in high-throughput genome editing experiments, since the selection of sgRNAs is often designed in regions with rare or even no restriction sites, and the mutation rate can be limited. Therefore, it is important to consider alternative methods for the detection of induced mutations through genome editing, regardless of the target site and/or the mutation rate. For this reason, the utilization of Sanger and/or next-generation sequencing (NGS) approaches is preferred [74,75]. Sequencing the target site guarantees that any successful mutations will be detected.

23. Amplify the target sequence using primers flanking the induced mutation site using a proofreading polymerase.

*Note*: Perform agarose electrophoresis and elute the expected size amplicon from the gel using a gel extraction kit or perform a PCR clean-up if there are no other PCR byproducts.

24. Submit the eluted (or purified) amplicon(s) for Sanger sequencing.
25. Analyze the sequencing results using Synthego’s free bioinformatics tool, Inference of CRISPR Edits (ICE) [76], to easily assess the possibility of putative CRISPR-induced edits at the target sites.
26. Further verification of the putative edits can be performed by TOPO-cloning of the eluted (or pure) amplicon(s) and subsequent submission of at least five clones for Sanger sequencing. This ensures that all possible variants are properly characterized, including heterozygous and biallelic loci.

*Note*: We recommend that submission of ≥ five clones per PCR amplicon suffice for the detection of potential edits using Sanger sequencing. For more accurate results, it is advisable to submit more clones [69] or to conduct multiplex target amplicon sequencing (see step 28).

**Figure 3:**
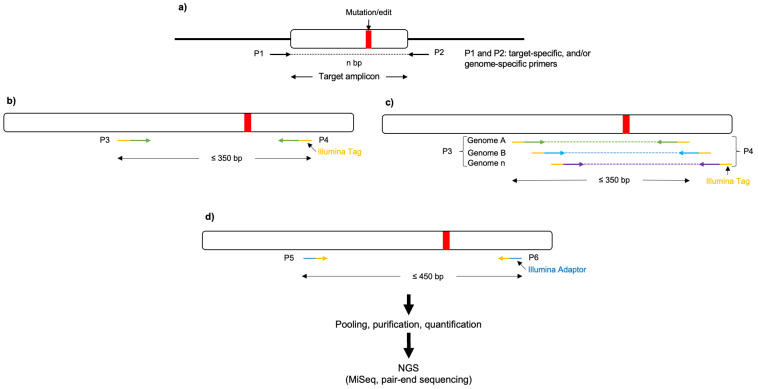
Schematic representation of a three-stage target amplicon workflow for the next-generation sequencing of haploid and polyploid crop species, based on the 16S Metagenomic Sequencing Library Preparation. (**a**) Forward and reverse primers (P1 and P2, respectively) flanking the region of interest are used to amplify templates from the genomic DNA (in polyploid crops P1 and P2 could serve as genome-specific primers). (**b**,**c**) Nested PCR is performed, using the eluted/purified amplicon from the previous step as a template, to incorporate Illumina overhang adapters into the primers (P3 and P4) for the respective genomes. (**d**) Additionally, nested PCR enables the addition of multiplexing indices and Illumina (San Diego, CA, USA) sequencing adapters (P5 and P6; https://support-docs.illumina.com/SHARE/AdapterSeq/illumina-adapter-sequences.pdf (accessed on 1 September 2023)). The generated libraries are then normalized, pooled, and sequenced on the MiSeq system.

27. Compare the sequencing results by mapping them to the template sequence (see step 3).
28. (Optional) Perform next-generation sequencing (NGS) of the target amplicons using the eluted (or pure) amplicon obtained previously (step 27) as the initial template (nested PCR) to determine the rate of induced mutations generated by CRISPR/Cas and potential off-target edits (Figure 3). To be cost-effective, this requires many amplicon samples to be pooled together using barcoded DNA indices to label each sample during the sequencing library preparation.

*Note*: A three-stage target amplicon workflow for the next-generation sequencing of haploid and polyploid crop species, based on the 16S Metagenomic Sequencing Library Preparation, can be used [77,78].

In our case, a pair-end target amplicon analysis (one million read pairs (with a Read Length of 250 × 250)) was performed using Illumina MiSeq, with either 48 or 96 barcoded samples per run.

All the PCR steps involved in the preparation of NGS libraries were performed using KAPA HiFi DNA Polymerase (Roche, Indianapolis, IN, USA), as has been suggested previously [79]. However, it has come to light recently that Quantabio RepliQa Hifi Toughmix produces the best outcomes for a variety of genomes and applications [80].

This approach is mainly suitable for the identification of unintended on-target modifications that have been introduced within the immediate vicinity of the target site [81]. A comprehensive assessment of off-target edits and other unintended modifications that could be present in genome-edited plants would require a different approach [82].

29. Analysis of the obtained Illumina MiSeq results using CRIS.py [83], a versatile and high-throughput analysis program for CRISPR-based genome editing.

## 3. Conclusions and Future Perspectives

Implementing these step-by-step guidelines should improve the rate of success for routine CRISPR/Cas9 (or other Cas nuclease) genome editing experiments in most major crops. Devoting time in the initial stages to carefully guide RNA design and validation is essential to ensure good results, especially for crops that have a lengthy in vitro tissue culture and regeneration process, where taking shortcuts early on can lead to disappointment at the end. At the same time, sgRNAs can fail for various reasons, so it is still prudent to have backup designs, such as targeting two nearby sgRNAs to knock out a target gene, so if one fails, there is still a good chance of knocking out the gene function with the single remaining guide RNA. Another bottleneck is *Agrobacterium*-mediated transformation and in vitro regeneration [84], since several crop species are difficult to transform and are often very slow to regenerate, such as cotton, which can take 18–24 months. As several research groups around the world are exploring alternative *in planta* transformation approaches, it is hoped that this bottleneck will be resolved over the next few years. Once a truly high-throughput transformation system is in place, this will enable genome editing techniques that are currently too difficult to be performed routinely, such as allele replacements using homology-directed repair. Once genotype-independent transformation and regeneration become feasible, genome editing can extend beyond the use of transformation-ready model genotypes and can be more readily applied to elite breeding materials. Applying genome editing to elite breeding materials could accelerate the development of edited plants for agricultural use, but necessitates careful assessment of the off-target modifications and unintended transgenic insertions in the resulting plants [8,9,10].

## Figures and Tables

**Table 1:** A list of online annotated databases for different crop species that can serve as repositories for identifying and obtaining the required sequence information for the respective target(s).

Crop Species	Web Links (All Links Were Accessed on 1 September 2023)
Rice	http://rice.uga.edu/ https://rapdb.dna.affrc.go.jp/
Wheat	http://wheat-urgi.versailles.inra.fr/Seq-Repository/Assemblies https://plants.ensembl.org/Triticum_aestivum/Info/Index https://wheat.pw.usda.gov/GG3/
Barley	https://wheat.pw.usda.gov/GG3/ https://ics.hutton.ac.uk/barleyrtd/index.html http://plants.ensembl.org/Hordeum_vulgare/Info/Index
Maize	https://www.maizegdb.org/ https://www.plantgdb.org/ZmGDB/
Sorghum	https://www.plantgdb.org/SbGDB/ https://plants.ensembl.org/Sorghum_bicolor/Info/Index https://www.sorghumbase.org/
Solanaceae	https://solgenomics.net/
Cotton	https://cottonfgd.net/ https://www.cottongen.org/
Soybean	https://soybase.org/
Legumes	https://www.legumeinfo.org/

**Table 2:** A list of online databases that are commonly used for the design of sgRNAs in different crop species.

Online Databases	Web Links (All Links Were Accessed on 1 September 2023)
CRISPR-P 2.0	http://crispr.hzau.edu.cn/CRISPR2/
CRISPR-direct	https://crispr.dbcls.jp
Chopchop	https://chopchop.cbu.uib.no
Target design	http://skl.scau.edu.cn/targetdesign/
CRISPR-PLANT v2	http://omap.org/crispr2/

## Data Availability

The data presented in this study are available within the published article.
